# Allele Distributions at Hybrid Incompatibility Loci Facilitate the Potential for Gene Flow between Cultivated and Weedy Rice in the US

**DOI:** 10.1371/journal.pone.0086647

**Published:** 2014-01-28

**Authors:** Stephanie M. Craig, Michael Reagon, Lauren E. Resnick, Ana L. Caicedo

**Affiliations:** 1 Biology Department, University of Massachusetts, Amherst, Massachusetts, United States of America; 2 Department of Evolution, Ecology, and Organismal Biology, Ohio State University, Lima, Ohio, United States of America; The University of Queensland, St. Lucia, Australia

## Abstract

The accumulation of independent mutations over time in two populations often leads to reproductive isolation. Reproductive isolation between diverging populations may be reinforced by barriers that occur either pre- or postzygotically. Hybrid sterility is the most common form of postzygotic isolation in plants. Four postzygotic sterility loci, comprising three hybrid sterility systems (*Sa, s5, DPL*), have been recently identified in *Oryza sativa.* These loci explain, in part, the limited hybridization that occurs between the domesticated cultivated rice varieties, *O. sativa* spp. *japonica* and *O. sativa* spp. *indica*. In the United States, cultivated fields of *japonica* rice are often invaded by conspecific weeds that have been shown to be of *indica* origin. Crop-weed hybrids have been identified in crop fields, but at low frequencies. Here we examined the possible role of these hybrid incompatibility loci in the interaction between cultivated and weedy rice. We identified a novel allele at *Sa* that seemingly prevents loss of fertility in hybrids. Additionally, we found wide-compatibility type alleles at strikingly high frequencies at the *Sa* and *s5* loci in weed groups, and a general lack of incompatible alleles between crops and weeds at the *DPL* loci. Our results suggest that weedy individuals, particularly those of the SH and BRH groups, should be able to freely hybridize with the local *japonica* crop, and that prezygotic factors, such as differences in flowering time, have been more important in limiting weed-crop gene flow in the past. As the selective landscape for weedy rice changes due to increased use of herbicide resistant strains of cultivated rice, the genetic barriers that hinder *indica-japonica* hybridization cannot be counted on to limit the flow of favorable crop genes into weeds.

## Introduction

Population divergence, a critical step in the process of speciation, is often accompanied and reinforced by the evolution of reproductive isolating mechanisms, which can occur pre – or postzygotically. In plants, hybrid sterility is the most common form of postzygotic isolation [1]. Hybrid sterility is thought to evolve according to the Bateson-Dobzhansky-Muller (BDM) theory of speciation, which posits that independent mutations occurring in diverging populations become fixed and then interact negatively in the background of the hybrid [2].

The cultivated rice complex (*Oryza sativa* L.) affords a rare opportunity to investigate the evolutionary history and underlying genetics of traits influencing postzygotic barriers to hybridization. The sterility observed in crosses of two subspecies of Asian cultivated rice, *O. sativa indica* and *japonica*, is one of the most extensively studied of all hybrid incompatibilities in plants [3]–[6]. *Indica* and *japonica* cultivars were domesticated ∼10,000 years ago and differ in various morphological characteristics and in their responses to a multitude of biotic and abiotic stresses [5]. Gene exchange between these rice subspecies would be highly beneficial to rice breeding practices, however, full exploitation of hybrid rice is limited by the tendency of hybrids to exhibit some degree of sterility [7], which can vary from 5 to 95% depending on the cross [8]. The identification of sterility-causing loci between these cultivars and subsequent determination of their contributions to sterility has been a topic of much research. Despite the ∼57 hybrid-incompatibility quantitative trait loci (QTL) detected so far in rice [9], only a few have been cloned and subjected to experimental testing. Four such loci, encompassing three hybrid sterility systems, are *Sa*, *s5*, and *DOPPELGANGER1* (*DPL1*) and *DOPPELGANGER2* (*DPL2*). *Sa* and *DPL1/2* affect pollen viability and *s5* sterility results in embryo-sac abortion [10]–[12].

All three of these hybrid sterility systems have been shown to cause semi-sterility in hybrids between *indica* and *japonica* cultivars, and could be partial contributors to the low levels of gene flow observed between these two rice subspecies. However, the possible roles of these loci in influencing gene flow between these main rice varieties and other *Oryza* groups have not been explored. There is potential for gene flow between *indica* and *japonica* cultivars and other Asian rice cultivars (e.g. *aus*, *aromatic*), the wild ancestor of cultivated rice (*O. rufipogon*), and the conspecific weed of cultivated rice known as weedy or red rice (*O. sativa*). Red rice is a troublesome weed that invades cultivated rice fields worldwide and displays competitive traits such as dormancy, high shattering, and rapid growth [13]. Weedy rice infestations can lead to a reduction of rice yields and considerable financial losses [14]. Gene flow between cultivated and weedy rice can have very negative agricultural consequences, such as the potential for crop traits to escape into weedy rice populations and unfavorable weedy traits contaminating seed stocks.

While weedy red rice is a worldwide problem of rice agriculture [13], the evolutionary origins of weedy rice and its relationship with the local rice crop differs throughout the world [15]–[17], affecting expectations of the potential for gene flow. In the US, local cultivated rice belongs to the *tropical japonica* variety of the *japonica* subspecies, while weedy rice is related to the *indica-aus* lineage. Two main genetically differentiated populations of red rice are known to co-occur in rice fields in the US: the most common straw hull (SH) group, which is characterized by straw colored grains, and the black hull awned (BHA) types, which typically have black colored grains with awns [16]. Studies of polymorphism have shown that the SH and BHA weedy groups are most closely related to, and likely descendant from, the *indica* and *aus* cultivated varieties, respectively [16], [18]. *Indica* and *aus* are closely related crop varieties, typically grown in lowland tropical regions of Asia, and distinct from the *tropical japonica* cultivars widely cultivated in the US. Further population structure is observed in the BHA group, which can be partitioned into two subpopulations (BHA1 and BHA2). Additionally, hybridization between SH and BHA groups has given rise to a group of weeds known as the BRH (brown hulled) group that occurs at lower frequency [16].

Although weedy rice is classified as the same species, is interfertile with, and co-occurs in fields of cultivated rice, weedy rice in the US shows limited hybridization with the local *japonica* crop [19]. Some hybrids between the local US *japonica* crop and SH or BHA weeds have been identified [16], as well as some evidence for past introgression [20] however there is little genetic evidence for extensive crop-weed hybridization. This may be due to self-pollination tendencies of cultivated and weedy rice [21] or differences in flowering time between crops and weeds in the field [13] (e.g. prezygotic mating barriers). However, since *indica* and *japonica* cultivars have been shown to experience limited hybrid compatibility due to various deleterious genetic interactions, it is possible that similar hybrid barriers limit the amount of outcrossing between weedy rice and the local *japonica* cultivar.

In this study we examine the allelic diversity of the characterized rice hybrid sterility loci (*Sa, s5*, and *DPL*s) in US weedy rice populations. We find very few barriers to intercrossing between weedy and cultivated rice at these loci, including the near fixation of a rare allele at the *Sa* locus, which seemingly confers wide-compatibility to some populations of weedy rice. Despite current low frequencies of hybrids in US rice fields, our results suggest that no postzygotic barriers should prevent widespread gene flow between weedy and cultivated rice.

## Materials and Methods

### Plant Material

Diverse *Oryza* seeds obtained from the United States Department of Agriculture (USDA), the International Rice Research Institute (IRRI), collections contributed by Dr. David Gealy of the Dale Bumpers National Rice Research Center, and Susan McCouch of Cornell University (Table S1) were grown at the University of Massachusetts Amherst. Our panel consisted of 107 individuals from multiple *Oryza* species, including weedy rice (51), *O. rufipogon* (25), *O. nivara* (2) and various *O. sativa* cultivars including *aus* (7), *indica* (10), and *japonica* (10); this latter group contained both US and Asian cultivars. Other AA genome *Oryza* species, *O. meridionalis* (1) and *O. glaberrima* (1), were included as outgroups (Table S1). The weedy groups used in our panel were previously defined by Reagon *et al.* [16] based on 48 sequence tagged site (STS) markers, and consisted of the main weedy groups in the US that have putative *indica* and *aus* ancestry (SH, BHA1 and BHA2), the BRH group believed to be a hybrid between SH and BHA weeds, and rarer weedy individuals classified as MX, which are likely early generation hybrids between the main weedy groups and the local *japonica* crop. Twenty *aus* and thirty *indica* individuals obtained from USDA Genetic Stocks – *Oryza* Collection (GSOR) and IRRI were later added to our survey for further genotyping at the *s5* and *SaF* loci (Table S2).

### Sequencing and Genotyping

DNA was extracted from leaf material of all accessions using a CTAB method. Primers were designed using Primer3 [22] to amplify portions of each hybrid sterility locus, taking into account previously described causal polymorphisms at each locus [10]–[12] (Table S3). *Indica* or *japonica* type alleles were genotyped for all individuals at each locus using either DNA sequencing, when alleles were differentiated by a single nucleotide polymorphism (SNP), or differential gel migration, when alleles could be visualized by a size difference due to insertion-deletions (indels). DNA sequences were aligned and edited using BioLign Version 2.09.1 (Tom Hall, NC State University). DNA sequences obtained were deposited into GenBank as population datasets under accession numbers KF892880–KF893259.

### Genetic Diversity and Phylogenetic Analysis

Summary statistics for each sequenced locus were obtained with DnaSP version 5.0 [23]. Statistics included Watterson’s estimator of nucleotide variation (θ_W_), the average pairwise nucleotide diversity (π) [24] and Tajima’s D (TD) [25]. Summary statistics for each locus were compared against 48 genome-representative STS loci [16] for outlier behavior. Heterozygotes were phased using the haplotype subtraction method [26]. Genealogical relationships among sequenced haplotypes at each locus were determined with Neighbor-Joining analyses using a Kimura-2-parameter model in MEGA5 [27]. For all loci, the Nipponbare sequence, the *temperate japonica* accession with a sequenced genome, was included as haplotype 1. Weedy accessions were examined for novel alleles, clade membership expectations based on known ancestry, and the likelihood of introgression with US cultivars based on genotypes at each locus. For simplicity, in the remainder of the manuscript the term “haplotype” is used to refer to the DNA sequence content of a given allele, while “allele-type” refers to the functional classification of an allele as *indica*-type, *japonica*-type, or (in some cases) wide compatibility-type (Table S4 and Table S5).

### Crosses and Quantifying Pollen Viability

Crosses were performed between *Oryza* accessions to compare pollen production among individuals with different *Sa* genotypes. Parents were planted in January 2012 and were grown in a walk-in Conviron PGW36 growth chamber at the University of Massachusetts Amherst under 11 hour days at 25°C. Panicles newly emerged from the boot, but not dehisced were chosen as the female. The top of each floret was cut off and the anthers removed with forceps, leaving the stigma intact; 20–30 florets were cut per panicle. A panicle on the verge of dehiscence was chosen to be the male parent. Both male and female panicles were placed in a single glycine bag and secured with a paperclip. Bags were collected after one month to check for hybrid seed. F_1_ seeds were heat-treated overnight at 34°C and then either plated on a petri dish or planted in soil. Heterozygosity of the F_1_ was confirmed *via* PCR.

Both homozygous parents of various *Sa* genotypes, as well as hybrid offspring were examined for pollen quality. For each sample, pollen grains of six anthers, from six different florets were suspended in 100 µl of Lugol’s Solution (LS), and then serially diluted in 90 µl of LS. Pollen viability was quantified by obtaining a nonviable-to-viable ratio of pollen observed under a Leica MZ 16FA microscope using LS, a potassium iodide stain that reacts with starch in viable cells dying them black; non-viable cells do not stain and appear clear. Three ratios were calculated using ImageJ (http://rsb.info.nih.gov/ij) within a fixed area from three fields of view, and averaged for percent viable pollen.

The quantity of pollen produced was measured using a 0.5 mm deep Nageotte Bright Line Hemacytometer, resulting in a pollen grain/µl concentration. Three measurements were obtained per individual and averaged to calculate mean pollen quantity.

## Results

### The Genealogy of *s5*

*S5* encodes an aspartic protease on chromosome six that is expressed in ovule tissue. Three allele types have been described: an *indica*-type (*s5-i*), a *japonica-*type (*s5*-*j*) and a wide compatibility allele (*s5*-n) [10]. SNPs C282A and C877T differentiate *s5-i* and *s5-j* alleles, while a 136 bp deletion in the N-terminus of *s5*-n renders it non-functional and confers wide-compatibility [10]. The dimerization of *s5-i* and *s5-j* causes embryo sac abortion and has been reported to reduce spikelet fertility by 46% [10].

**Figure 1:**
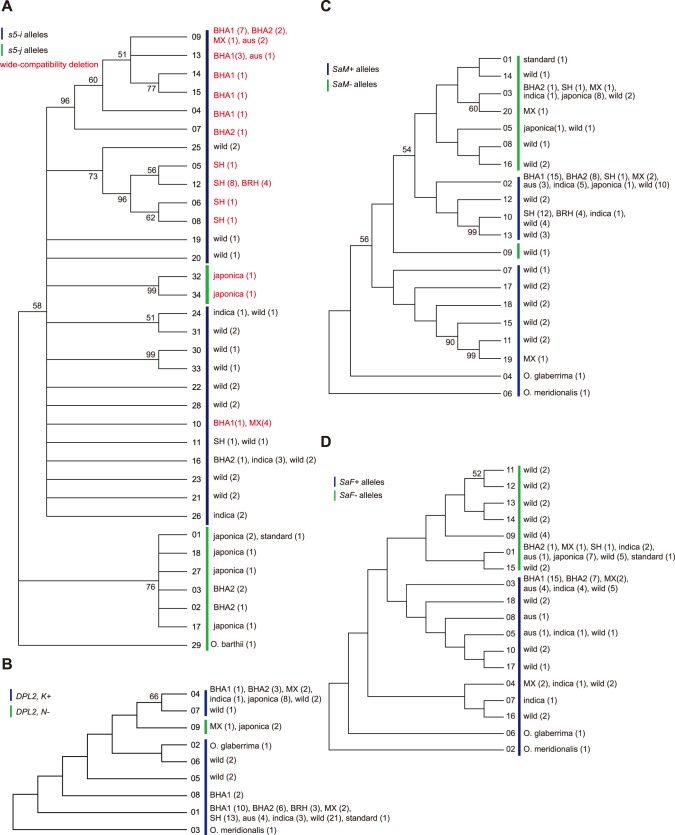
Neighbor joining trees of hybrid-incompatibility loci sequenced haplotypes. Numbers on branches correspond to bootstrap percentages from 500 replicates. Bootstrap values below 50 are not shown. Tip labels correspond to haplotype numbers as in Table S4, and to *Oryza* groups in which the haplotype was found. Numbers in parentheses correspond to the number of alleles found for each haplotype. Green bars designate haplotypes reported as typically from the *japonica* group, and blue bars designate haplotypes typical of the *indica* group. A. Haplotype tree for *s5.* B. Haplotype tree for *DPL2.* C. Haplotype tree for *SaM.* D. Haplotype tree for *SaF.*

We sequenced a total of 1897 bp for *s5*, beginning 949 bp downstream of the start codon in the second exon to 1016 bp after the stop codon, in samples of weedy, wild and cultivated rice. Since sequencing of outgroups failed repeatedly for this locus, we included the published sequence of an *O. barthii* (Gen Bank Accession # JF298922). Thirty-five SNPs and five indels were identified within this region encompassing thirty-four haplotypes (Figure 1A; Table S4, Table S5A). We classified alleles as either *indica*- or *japonica-*type based on the SNPs reported by Chen *et. al* [10]. *Indica*-type alleles were further grouped based on whether they contained the wide-compatibility deletion (Table S4).

Previous analysis of the *s5* locus in wild groups found the majority of wild accessions to carry *indica*-type alleles [28]. Likewise, 97% of wild alleles in our study (29/30) were *indica*-type (Table 1). Du *et al.* [28] also found *indica* and *japonica*-type alleles fixed within their respective groups, although the *aus* group was not explicitly characterized. We found that all *indica* and *aus* individuals in our panel carried *indica*-type alleles, while only 62.5% (5/8) of *japonica* individuals carried the expected *japonica* allele types (Table 1). The wide-compatibility deletion was detected in 50% of cultivars possessing *indica*-type alleles, including one grown in the US (sus02), and in 14% of wild *indica*-type alleles (Figure 1A, Table 1, and Table S4). Interestingly, no *indica* individuals surveyed possessed the wide-compatibility deletion, but all *aus* individuals did (Figure 1A, Table 1).

**Table 1:** Frequency (in percentage) of allele types found at each population and each hybrid incompatibility locus in our core set of accessions.

			*Oryza* group									Total
Locus		Allele Type	*indica*	*aus*	*japonica*	SH	BHA1	BHA2	MX	BRH	Wild	samples
***s5***		***s5-i***	100	100	37	100	100	63	100	100	97	
		**wc#**	0	100	100	86	100	80	100	100	14	
		***s5-j***	0	0	63	0	0	37	0	0	3	
	**alleles sampled** *		6	3	8	14	14	8	5	4	30	92
***DPL1***		***DPL1-K−***	67	100	21	0	77	100	0	25	44	
		***DPL1-N+***	33	0	79	100	23	0	100	75	56	
	**alleles sampled**		6	5	14	11	13	7	5	4	32	97
***DPL2***		***DPL2-K+***	100	100	85	100	100	100	80	100	100	
		***DPL2-N−***	0	0	15	0	0	0	20	0	0	
	**alleles sampled**		4	4	13	13	13	9	5	3	28	92
***SaM***		***SaM+***	89	80	10	13	100	89	80	20	66	
		***SaM−***	11	20	90	6	0	11	20	0	18	
		***SaM+X***	0	0	0	75	0	0	0	60	16	
		***SaM-X***	0	0	0	6	0	0	0	20	0	
	**alleles sampled**		9	5	10	16	15	9	5	5	38	112
***SaF***		***SaF+***	78	86	0	0	100	88	80	0	39	
		***SaF−***	22	14	100	7	0	12	20	0	43	
		***SaFX***	0	0	0	93	0	0	0	100	18	
	**alleles sampled**		9	7	8	14	15	8	5	4	44	114

# wc = wide compatibility; wide compatibility percentages are out of total *indica*-type alleles.

* Due to differences in mating system, *O. rufipogon* genotypes are considered diploid; all other samples are considered as haploid, except in cases of rare heterozygotes.

As expected based on their putative ancestry, all weedy BHA1, SH, and BRH individuals examined possessed *indica*-type alleles (Table 1, Table S4). All MX weeds also possessed *indica*-type alleles, despite their mixed weed x *japonica* ancestry. The only weedy group not fixed for *indica*-type alleles was BHA2, in which 37% (3/8) of the individuals carried *japonica*-type alleles (Table 1 and Table S4).

Despite the wide occurrence of *indica*-type alleles, not all sequenced weedy haplotypes were identical to those detected in cultivars. Nine novel haplotypes were detected in the weedy populations, (Haplotypes 2–8, 14 and 15) (Figure 1A). These haplotypes are not shared with wild or cultivated individuals in our panel, and, except for haplotype 2 [28], have not been previously reported in the literature. Remarkably, not a single SH weed sequenced carried an identical haplotype to any of the *indica*, its putative progenitor group, in our panel (Figure 1A, Table S4).

Regardless of population, most weeds (87%) contained the wide compatibility deletion (*s5-n* allele; Figure 1A, Table 1). This deletion occurred in only six of our cultivated individuals, all from the *aus* and *japonica* cultivar groups, and, unlike their putative *indica* progenitors, SH weeds often have the deletion. Based on this finding, we further explored the possible origins of *s5-n* by genotyping an additional 20 *aus* and 30 *indica* accessions from south Asia (Table S2). The deletion was very common in *aus*, detected in 19/20 individuals. The deletion was rare in *indica* occurring in only six individuals, mostly from Nepal. The widespread presence of the wide-compatibility allele in US weedy rice groups suggests that this locus poses no postzygotic barrier to hybridization with US crops or hybridization between weedy groups.

### Genealogy and Allelic Distribution at the *DPL* loci

*DPL1* (chromosome 1) and *DPL2* (chromosome 6) are paralogous hybrid incompatibility genes that encode small plant proteins and are highly expressed in mature anthers [12]. *Japonica* cultivars have been described as containing a functional copy of *DPL1* (*DPL1*-*N+*), and a non-functional allele of *DPL2* (*DPL2-N−*) due to a SNP at A434G [12]. *Indica* and *aus* cultivars have been described as carrying a non-functional allele of *DPL1* (*DPL1*-K−) due to a 517 bp insertion 204 bp downstream of the start codon, and a functional copy of *DPL2* (*DPL2*-K+) [12]. Pollen carrying non-functional alleles at both loci (e.g. *DPL1*-K− *DPL1*-K−//*DPL2-N− DPL2-N−*) is non-viable [12].

#### Allelic Distribution of *DPL1*

*DPL1* was genotyped for functionality based on the presence/absence of the 517 bp insertion (Mizuta *et. al* 2010). Individuals without the insertion were categorized as functional (*N+*), and individuals with the insertion were classified as non-functional (*K−*) (Table S4). Previously, the presumably ancestral functional *DPL1* alleles were found to be more prevalent in wild rice populations [12]. Similarly, we found *DPL1-N+* alleles were present at higher frequencies in our *O. rufipogon/nivara* sample (56%) (Table 1, Table S4). Within cultivated populations, Mizuta *et al.* [12] found both alleles at equal frequencies in *indica*; however we found the *DPL1*-*K*− allele at higher frequency than the *DPL1*-*N+* allele in *indica* (4/6), and fixed in all *aus* individuals surveyed (Table 1). Mizuta *et al*. [12] described the *japonica* group as fixed for *DPL1-N+* alleles. Unexpectedly, we found 21% of our *japonica* individuals to carry nonfunctional *DPL1-K*− alleles; however, among surveyed US *japonica* cultivars, all but one carried *DPL1-N+* alleles (Table 1, Table S4).

All BHA2 weedy individuals possessed the non-functional *indica*-type allele (*DPL1*-*K*−), which is consistent with their *aus* ancestry. However, three BHA1 individuals, a group that also has *aus* ancestry, carried *DPL1*-*N+*, which we did not find in any *aus* individual. MX and SH populations only had *DPL1-N+* alleles, even though we found this allele at lower frequency within *indica*, and the BRH group also carried predominantly *DPL1-N+* alleles (Table 1, Table S4).

#### The Genealogy of *DPL2*

We sequenced 499 bp of *DPL2*, encompassing from 16 bp into the 1^st^ exon through 19 bp into the second exon of the gene. Four indels and 22 SNPs were found in our sample, encompassing a total of nine haplotypes (Figure 1B; Table S5B). *DPL2*-*K+* has previously been found to be more frequent in wild populations [12]. Accordingly, this allele was fixed within our wild accessions (Table 1; Table S4). In cultivated populations, the non-functional *DPL2*-*N*− and functional *DPL2-K+* alleles have previously been reported as fixed within the *japonica* and *indica* populations, respectively. The *DPL2-K+* allele was also fixed in our *indica* samples, but, strikingly, the *DPL2-N*− allele was rare in our *japonica* individuals, with only 15% (2/13) carrying *japonica*-like alleles (Table 1; Table S4). Moreover, only one of our US *japonica* individuals carried *DPL2-N*− (Table 1).

Similar to wild and domesticated groups, the *indica*-type allele (*DPL2*-*K+*) was nearly fixed in weedy groups (98%, 42/43). The only *japonica*-type allele was carried by one MX weed thought to be a putative hybrid between an SH weed and a *japonica* cultivar [16] (Table 1; Table S4). The haplotype carried by this weed, haplotype 9, is the only *japonica*-type haplotype we found in all our sequences, indicating low sequence diversity for *DPL2-N*− in contrast with the ancestral *DPL2-K+* alleles (Figure 1B). The remaining weedy groups fell within three haplotypes, 1, 4, and 8, with weedy individuals grouping with their domesticated progenitors in haplotypes 1 and 4 (Figure 1B). Haplotype 8, a novel haplotype that occurs in two BHA1 individuals, is characterized by a 1 bp deletion in the coding region of *DPL2*, which could lead to a non-functional gene.

#### The Distribution of *DPL* Genotypes

For simplicity, for hybrid incompatibility loci consisting of two genes, we will list genotypes in haploid format in the remainder of this manuscript; only in the case of hybrid progeny/heterozygotes will we write out the complete diploid genotype. Only two genotypes across both *DPL* loci were detected in our wild populations: *DPL1-K−/DPL2*-*K+* (42%) and *DPL1-N+*/*DPL2*-*K+* (58%) (Table S4). Typical *japonica* cultivars are reported to carry *DPL1-N+/DPL2- N*− [12], but the predominant genotype within our *japonica* panel was *DPL1-N+*/*DPL2-K+.* Only two of our *japonica* individuals (one from the US) possessed the genotype expected of typical *japonicas*, suggesting that previous characterizations of *DPL* loci may have been too narrow (Table S4). Mizuta *et al.* [12] found that half of the *indica* population had *DPL1-*K−/*DPL2-K*+ genotypes, similar to what we found in our *indica* panel (Table S4). This genotype was also fixed among our *aus* cultivars and was found in three *japonica* individuals within our panel (Table S4).

In weedy populations, the *DPL1-N+*/*DPL2-K+* genotype, with functional alleles at both loci, was fixed in SH, nearly fixed in MX and BRH individuals, and present in three individuals from the BHA1 group (Table S4). The *DPL1-*K−/*DPL2-*K+ genotype was fixed in BHA2 and present at high frequency (73%) in the BHA1 group (Table S4). Given the high frequency of the *DPL1-N+*/*DPL2-K+* genotype in US cultivars as well as several of the weed groups, the *DPL* loci do not seem to present a barrier to gene flow between cultivated and weedy rice in the US. Only crosses between *DPL1-*K− carrying weeds (primarily in the BHA groups) and the very rare *DPL2-N*− carrying cultivars (only 1 out of 8 surveyed cultivars possessed this allele type), would be expected to result in the *DPL1-K−/DPL2-N*− sterility-causing genotype.

### Genealogical Relationships at the *Sa* Locus

The *Sa* locus comprises two adjacent genes on chromosome 1, *SaM* and *SaF*. *SaM* encodes a small ubiquitin-like modifier E3 ligase-like protein and *SaF* encodes an F-box protein [11]. *Indica* cultivars typically possess a *SaM*+/*SaF*+ genotype, while *japonica* cultivars have a derived *SaM*−/*SaF*− genotype. A *SaM* heterozygote and a *SaF*+ allele are required to cause male semi-sterility (usually about 50%; [11]). *SaM*+ and *SaM−* are differentiated by a G-to-T polymorphism in the fifth intron, resulting in a truncated protein [11]. SaF+ and SaF− are differentiated by a C-to-T transition at position 287 that leads to an amino acid change. Mechanistically, during the uninucleate stage of gamete development, direct interaction of SaF+ with SaM*−* causes selective abortion of SaM*−* bearing microspores [11].

#### The Genealogy of *SaM*

We amplified a 634 bp portion of *SaM* starting four bp into the 4^th^ exon through 44 bp downstream of 5^th^ exon. Within this portion of the gene, we found seven indels and 20 SNPs, distributed among 20 haplotypes (Table S5C). Alleles were further classified as either *indica* (*SaM+*) or *japonica* (*SaM−*)*-*like based on differentiating polymorphisms assigned by [11] (Table S4). Consistent with two previous studies [11], [29], we found 82% of wild alleles in our panel were *SaM+* (Table 1;Table S4). The *SaM+* allele has also been documented as the most common in *indica* cultivars [11], [29]. Likewise, 89% of our *indica* individuals carried the *SaM+* allele (Figure 1C, Table 1). The *SaM−* allele is nearly fixed in all *japonica* populations reported to date [11], [29]. Consistently, we found only one *japonica* individual (str02) with a *SaM+* allele in our sample. Consistent with its close relationship with *indica*, we found that four of five *aus* individuals had the *SaM+* allele (Table 1; Table S4).

As expected based on US weed ancestry, most of our weed alleles (90% or 45/50) were *SaM+*. Only four individuals from several weedy groups (BHA2, SH, and MX) possessed the *SaM−* allele (Table 1; Table S4). Weeds largely possessed haplotypes identical to those in progenitor groups or other cultivated groups (Figure 1C). Two novel haplotypes were observed in the MX groups (19 and 20) (Figure 1C).

#### The Genealogy of *SaF*

We amplified a 1.3 kb portion of *SaF* starting 674 bp into the first exon through 357 bp into the 3^rd^ exon. Repeated amplification failures were observed mostly in SH (15/16) and BRH (5/5) groups, so initial genotyping was carried out in the remaining *Oryza* groups (Table S4). Within this region, we found one indel and 24 SNPs and 18 different haplotypes (Figure 1D; Table S5D). Alleles were further classified as either *indica* (*SaF+*) or *japonica* (*SaF−*)*-*like based on differentiating polymorphisms assigned by [11] (Table S4).

Previous reports found wild populations to carry both allele types at relatively equal frequencies, which was consistent with our findings among individuals that amplified (Table 1). Twenty-two percent of our *indica* individuals had a *SaF−* allele, while previous reports found 10% of *indica* individuals carrying this *japonica*-type allele [11]. *SaF+* was nearly fixed within our *aus* panel (Figure 1D, Table 1; Table S4). *SaF−* was fixed in our *japonica* samples (Table 1; Table S4), consistent with previous studies [11], [29].

Among samples that amplified, we found that 90% of weedy individuals (26/29) carried *SaF+* alleles (Table S4). Consistent with their *aus* ancestry, *SaF+* is fixed in BHA1, and is found in 87.5% of BHA2 individuals (Table 1; Table S4). The majority of MX weeds also carry *SaF+* alleles (Table 1; Table S4). The three weedy individuals detected with *japonica*-type alleles fall into haplotype 1, the most frequent *japonica*-type haplotype, and also the only *japonica*-type haplotype we detected in *indica* cultivars (Figure 1D).

#### A Novel *SaF* Allele: *SaFX*

**Figure 2:**
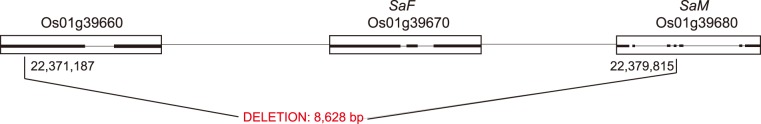
Diagrammatic representation of the 8,628*SaFX* deletion. The deletion begins within the gene upstream of *SaF*, a putative transposon protein, and knocks out the first four exons of *SaM*.

Early attempts to sequence the *SaF* gene were complicated by amplification failures for the majority of samples belonging to the SH and BRH weedy groups and a few other *Oryza* samples. We obtained the whole-genome sequence of a single SH individual (Young and Caicedo, unpublished information), and noted a large deletion spanning the entire *SaF* gene. To determine the exact genomic boundaries of the *SaF* deletion, we designed primers on both sides of the inferred deletion breakpoints (Table S3) and amplified and sequenced four SH samples. We found that the deletion spans 8,628 bp (from coordinates 22,371,187–22,379,815 of chromosome 1 in the MSU 6.0 rice genome) and begins 2,902 bp upstream from the start of *SaF*. This deletion partially knocks out the gene *Os01g39660,* a putative transposon protein, as well as the first four exons (1,240 bp) of *SaM*, the gene located immediately downstream of *SaF* (Figure 2).

We found no evidence of this deletion having been reported before, and therefore named the resulting *SaF* deletion allele *SaFX*. To genotype for the *SaFX* allele, a complementary set of primers that amplified specifically only in the presence (SaFdel primers) or absence (SaF primers) of the deletion was used (Table S3). We found that 61% of individuals that failed amplification for *SaF* (20/33) amplified with our *SaFX* primers (Table 1; Table S4).

The boundary of the *SaFX* deletion extends four exons into *SaM*, but prior to the *SaM+/SaM−* differentiating SNP in the fifth intron (Figure 2). Taking this into account, we re-assigned *SaM* alleles in individuals containing *SaFX* as either *SaM+X* or *SaM-X*, to indicate which type of allele it carries (*indica* (+) or *japonica* (−)) and that a large portion of the gene is missing (Table 1; Table S4). Only two individuals with *SaM-X* alleles were found (Table S4), and the deletion breakpoint seemed identical as in the *SaM+X* alleles.

Given the frequency of this deletion in the SH groups, we expected to find this deletion in both cultivated and wild groups, particularly in *indica*, which is believed to be ancestral to the SH weed group. While the deletion was found in six wild individuals (Table 1; Table S4), we found no individuals with the deletion among the *indica* included in our initial panel. We thus expanded our sample set to genotype the same 50 individuals belonging to the *aus* and *indica* cultivar groups that were genotyped for *s5*. The *SaFX* deletion was detected only in four *indica* individuals from Nepal (3) and India (1) and one *aus* cultivar from India, suggesting a low frequency of this allele in wild and cultivated *Oryza* groups (Table S2).

#### The Distribution of *Sa* Genotypes

Wild populations have previously been reported to carry primarily *SaM+*/*SaF+* genotypes [29]. Of the 36 wild samples with genotypes at both loci, 47% carried *SaM+*/*SaF+* and 14% carried *SaM−/SaF−* (Table 1; Table S4). However, we also detected novel allelic combinations of *SaM−/SaF+* (5%), *SaM+*/*SaF−* (17%) and *SaM+X*/*SaFX* (17%) (Table S4). We believe that *SaFX* arose in a *SaM+* background, since we found it only in combination with *SaM+X* alleles in wild individuals; however we did find the *SaFX*/*SaM-X* combination in two weeds (Table S4). Consistent with previous research [11] the *SaM−/SaF−* genotype was nearly fixed in our *japonica* individuals, including the US cultivars surveyed (Table S4). Likewise, the typical *indica* genotype, *SaM+/SaF+,* was found to be nearly fixed in our *indica* and *aus* panels as well (Table S4).

Consistent with *aus* ancestry, *SaM+*/*SaF+* is fixed and nearly fixed in BHA1 and BHA2, respectively. Given the common occurrence of *SaM−/SaF−* genotypes in US cultivars, interactions at this locus could limit hybridization with BHA weeds. *SaM+X*/*SaFX* was nearly fixed in the BRH and SH groups. However, we were not able to predict how *SaFX* affects hybrid sterility levels in crop-weed hybrids since this is the first report of this allele. We speculate that *SaFX* is a non-functional allele since it knocks out the entire *SaF* gene and four exons of *SaM* (see below).

### The Consequences of *SaFX* on Pollen Viability

Because the *SaFX* gene has not been reported in the literature, we attempted to assess the phenotypic consequences of carrying this allele on pollen production. We designed crosses to test the phenotypic consequences of *SaFX* in various genetic backgrounds (Table 2). We quantified both the quantity and quality of pollen produced in parental individuals and in crosses performed between these parentals.

**Table 2:** Successful crosses carried out to assess the effects of the *SaFX* allele.

Cross	Parental 1genotype	Parental 1accession	Parental 2genotype	Parental 2accession	F1 genotype
1	*SaM+X*/*SaFX*	rr07	*SaM−/SaF−*	rr06	*SaM+X*/*SaM−/*/*SaFX*/*SaF−*
2	*SaM-X*/*SaFX*	rr15	*SaM+*/*SaF−*	or18	*SaM-X*/*SaM+*//*SaFX*/*SaF−*
3	*SaM-X*/*SaFX*	rr33	*SaM+*/*SaF+*	rr03	*SaM-X*/*SaM+*//*SaFX/SaF+*

We assessed five common parental genotypes: *SaM+/SaF+, SaM+/SaF−*, *SaM−/SaF−*, *SaM+X/SaFX*, and *SaM-X/SaFX*, which were present within our panel. Ideally, our goal was to have each genotype be both the male and female parent, but varied flowering dates prevented us from making all intended crosses (Table 2). The resulting, verified hybrid genotypes we obtained are shown in Table 2.

#### Pollen Quality

Non-viable pollen ratios obtained for each individual are shown in Table 3. The parental individual with the highest non-viable ratio possessed a *SaM-X/SaFX* genotype with an average of 44% non-viable pollen (Table 3). However, another individual with the same genotype only had 6.8% non-viable pollen. The homozygous genotype that produced the most viable pollen was *SaM+/SaF−* with an average of 5.6% non-viability, but variance was high among individuals within the same genotype (Table 3). We performed a t-test comparing the parental individuals with a *SaFX* allele to those without *SaFX* and found no effect of *SaFX* on pollen quality (*P* = 0.9).

**Table 3:** Pollen viability in individuals with different *Sa* genotypes.

Genotype#	Accession	Average %Non-Viablepollen*	GenotypeAverage(sd)∧
*SaM+/SaF+*	rr03	8.4	
	sin02	6.1	
	rr39	8.8	
			7.7 (6.5)
*SaM−/SaF−*	rr22	12.3	
	rr22	29.5	
	rr06	19	
	rr53	6	
			16.7 (14)
*SaM+/SaF−*	or18	8	
	sin08	3.2	
			5.6 (2.5)
*SaM+X/SaFX*	rr07	5.4	
	rr08	6.6	
	rr12	15	
	rr11	6	
			8.25 (5.4)
*SaM-X/SaFX*	rr33	6.8	
	rr15	44	
			25.4 (24.4)
*SaM+X/SaM−//SaF−/SaFX*	F1	13.1	
			13.1 (4.4)
*SaM+/SaM-X//SaF−/SaFX*	F1	15	
			15 (2)
*SaM+/SaM-X//SaF+/SaFX*	F1	5.3	
			5.3 (3)

# parental genotypes listed as haploid for simplicity. All parents are homozygous.

* Values are averages of three measurements per individual.

∧ Averages and standard deviations calculated from all original raw measurements; standard deviations are in parentheses.

*SaFX* does not seem to affect pollen quality in the resulting F_1_ hybrids, though our small sample size limits our statistical power. The typical sterility-causing genotype at *Sa*, a *SaM* heterozygote and at least one *SaF+* allele, usually leads to a 50% decrease in total pollen viability [11]. Such drastic decreases in pollen viability were not seen in any of our F_1_ (Table 3). Non-viable ratios among the progeny do not differ significantly from parental values (*P* = 0.35). Our hypothesis of *SaM* being non-functional due to the *SaFX* deletion is supported by the observation of no decrease in pollen quality in our *SaM+X*/*SaM−/*/*SaF+*/*SaFX* hybrid, as a functional *SaM* heterozygote and *SaF+* allele should have resulted in a substantial decrease in total pollen viability.

#### Pollen Quantity

We also determined if the amount of pollen produced was affected by the presence of a *SaFX* allele (Table 4). There was considerable variation in the amount of pollen produced among parents regardless of allele type (Table 4). The highest pollen-producing individual had a *SaM−/SaF−* genotype (97.5 pollen grains/µl) and the lowest was *SaM−/SaFX* (14.1 pollen grains/µl) (Table 4). We found no statistically significant differences in pollen production between parents with and without the *SaFX* deletion (t-test, *P* = 0.54).

**Table 4:** Pollen quantity in individuals with different *Sa* genotypes.

Genotype#	Accession	AveragePollenQuantity(grains/µl)*	GenotypeAverage(sd)∧
*SaM+/SaF+*	rr39	21.2	
	rr03	59.6	
			40.4 (22.2)
*SaM−/SaF−*	sus02	27.47	
	rr56	46	
	sus01	97.5	
			57 (37.6)
*SaM+/SaF−*	sin08	42.4	
			42.4 (11.4)
*SaM+X/SaFX*	rr12	26.5	
	rr07	28.5	
	rr11	37.5	
			30.8 (14.6)
*SaM-X/SaFX*	rr33	32.8	
	rr15	14.1	
			23.45 (11.2)
*SaM+/SaM-X//SaF−/SaFX*	F1	97.2	
			97.2 (20.3)
*SaM−/SaM+X//SaF−/SaFX*	F1	27.6	
			27.6 (13)
*SaM+/SaM-X//SaF+/SaFX*	F1	51.7	
			51.7 (1.2)

# parental genotypes listed as haploid for simplicity. All parents are homozygous.

* Values are averages of three measurements per individual.

∧ Averages and standard deviations calculated from all original raw measurements; standard deviations are in parentheses.

There was no obvious effect on pollen production in the hybrid progeny carrying the *SaFX* allele. Pollen production ranged from close to the highest observed in parents (*SaM+*/*SaM-X*//*SaF−/SaFX*), to among the lower values (*SaM−/SaM+X*//*SaF−/SaFX*) (Table 4). Hybrid pollen production did not differ significantly from the parents (*P = *0.44). As before, the *SaM+X*/*SaM−/*/*SaF+*/*SaFX* hybrid did not display any evidence of reduced pollen production, suggesting that a functional *SaM+* protein is not being produced due to the deletion.

### Summary Statistics

Genetic diversity statistics were calculated for all members of the main weedy populations, as well as *indica*, *japonica, aus* and wild populations at each locus (Table 5). We attempted to look for any common patterns in polymorphism trends across the three hybrid incompatibility loci.

**Table 5:** Summary statistics for sequenced loci.

		*Oryza* group						
Locus	Statistic	BHA1	BHA2	SH	*indica*	*Aus*	*japonica*	wild
***s5***	**π**	0.0013	0.004	0.0007	0.0005	0.0004	0.003	0.0021
	**θ**	0.002	0.003	0.0011	0.0005	0.0004	0.0022	0.0035
	**TD**	−1.396	1.846	−1.269	−0.05	n/a#	0.826	−1.385
***DPL2***	**π**	0.0003	0.0011	0	0.0011	0	0.0031	0.0033
	**θ**	0.0007	0.0008	0	0.0012	0	0.0031	0.0104
	**TD**	−1.149	0.986	n/a	−0.612	n/a	0.022	−**2.374** *
***SaM***	**π**	0	0.0014	0.0019	0.0035	0.002	0.0021	0.0072
	**θ**	0	0.002	0.003	0.0044	0.0023	0.0028	0.0074
	**TD**	n/a	−1.609	−1.183	−0.901	−1.048	−1.035	−0.096
***SaF***	**π**	0	0.0024	0	0.0012	0.0008	0	0.003
	**θ**	0	0.0004	0	0.0014	0.0018	0	0.0042
	**TD**	n/a	−1.055	n/a	−0.689	−1.358	n/a	−1.037
**STS** ∧	**π**	0.0007	0.0005	0.0006	0.0016	0.0012	0.0011	0.0044
	**θ**	0.0008	0.0006	0.0004	0.0017	0.0011	0.0014	0.0056
	**TD**	−0.177	0.042	−0.441	−0.026	0.092	−0.773	−0.729

# TD values only calculated when more then four sequences available.

* Bolded values indicate significant TD.

∧ based on averages from Reagon et. al 2010.

A previous study based on STS loci among *Oryza* and weedy groups and found wild populations to harbor the most genetic diversity, followed by intermediate levels in the cultivars and low levels in US weedy groups, due to a genetic bottleneck upon US colonization [16]. In our study, wild populations were the most diverse, as expected, except at *s5* and *DPL2,* where BHA2 and *japonica* have the highest levels of nucleotide diversity (π), respectively (Table 5). Levels of diversity for hybrid incompatibility genes in weed groups varied among loci, but were sometimes an order of magnitude larger than the genome-wide averages. Particularly noticeable were the high levels of diversity observed in the BHA groups at *s5*. At this locus, all three weed groups surpassed the levels of diversity seen in their putative cultivated ancestors (Table 5); moreover, all weed groups possessed novel haplotypes not seen in cultivated or weed groups.

When polymorphism was present, TD values for many weed groups across all hybrid incompatibility loci tended to be more negative than genomic averages, indicating a tendency towards excess of rare mutations. At the *s5* locus, SH and BHA1 TD values are consistent with the occurrence of novel alleles. Also, noticeably, BHA2 at *s5* is associated with a very positive TD, which is consistent with the moderate frequencies of *indica* and *japonica*-type alleles in this group (Figure 1, Table 5).

## Discussion

### The Origin and Evolution of Hybrid Sterility Loci in US Weedy Groups

The demographic history of US weed groups based on random [16] and “weediness” candidate loci [20], [30] has been previously described, providing a framework for our expectations at hybrid sterility loci. Evidence to date suggests that the most common US weed groups, SH and BHA, which co-occur in crop fields, are directly descended from the *indica* and *aus* cultivated groups respectively. Additional low-frequency weedy groups are products of weed-weed hybridization (BRH) or hybridization with the local *japonica* crop (MX). US weeds tend to harbor very low levels of genetic diversity compared to wild and cultivated *Oryza* groups, due to a genetic bottleneck upon US colonization. The expectations based on genome-wide surveys, however, were not always borne out at hybrid incompatibility loci.

Levels of diversity among our weedy groups varied across hybrid incompatibility loci, but were sometimes an order of magnitude higher than the genome-wide averages (Table 5). Particularly striking were the high levels of variation observed at *s5* (Table 5). At this locus, all weed groups had greater levels of diversity than those seen in their putative cultivated ancestors and possessed novel haplotypes. It should be noted that we sequenced more noncoding sequence at this locus than the other hybrid incompatibility and STS loci. However, this cannot account for greater diversity within weed groups compared to their cultivated ancestors. A possible explanation is that having the wide-compatibility deletion, which makes the *s5* gene non-functional, has removed selection at this locus, allowing for higher levels of polymorphism in weedy groups. In the case of the BHA2 group, the high levels of diversity and positive TD could be due to hybridization, given the presence of several *japonica*-type alleles (Table S4, Table 1). No overall evolutionary trend was observed among hybrid sterility loci in the weedy groups, suggesting that each locus has been subjected to independent evolutionary forces.

As expected, the allele types found within weedy group were also largely found within their ancestral cultivated populations, albeit at varying frequencies (Table 1). However, the occurrence of occasional allele types within the main weedy populations that are not found in their putative ancestors, suggest possible hybridization. For example, several members of BHA1 carry *DPL1*-*N+* and *DPL2*-*K+* alleles, a genotype not observed in any of their putative *aus* ancestors. This could indicate hybridization with the local *japonica* crop, or with SH, BRH or MX individuals. As mentioned above, some BHA2 individuals carry *s5-j* alleles, suggesting possible hybridization with the local *japonica* crop.

For weed groups of known hybrid origin, contributions of each parental group vary at each locus. BRH, a weedy hybrid of SH and BHA, carries alleles common in both parental groups*;* however, at *DPL2* and *Sa*, the genotype found in the SH population is more common. The MX individuals, comprising hybrids of *japonica* cultivars and either SH or BHA weeds, carry alleles found in all three parental groups at *s5, DPL2*, and *Sa*. Curiously, however, *SaFX* is nearly fixed in SH but is not found in any MX individuals.

### Origins and Implications of the *SaFX* Allele

This is the first report of the *SaFX* allele, a deletion knocking out the *SaF* gene as well as portions of the *SaM* gene. We believe *SaFX* arose before domestication, as it was found in three individuals in our wild rice samples originating from Laos, Cambodia and Papua New Guinea (Table S4). However, it has remained at low frequency in both wild and cultivated populations, where it also seems to be geographically restricted. The *indica* and *aus* individuals we detected with the deletion are from Nepal and India (Table S4). The low frequency of this allele in wild and cultivated rice groups suggests lack of selection for the *SaFX* allele in these populations.

The low frequency of *SaFX* in wild and cultivated groups is in contrast to its near-fixation in the SH and BRH weedy groups. While further studies need to be done to evaluate how *SaFX* behaves between inter-subspecific crosses, our analyses suggest that *SaFX* has no significant effects on pollen quality or quantity. This deletion may counteract the sterility-causing interaction between a *SaM* heterozygote and *SaF+* allele, making *SaFX* comparable to the wide-compatibility allele at the *s5* locus in enabling gene flow between *indica* and *japonica* populations. The prominence of *SaFX* in some weedy groups may be a consequence of founder effects – perhaps SH weeds descend from *indica* cultivars from Nepal. This view is supported by the high frequency of *s5-n* alleles in the SH group, which was also only found in *indica* cultivars from Nepal (Table S2). However, it is also possible that selection may have favored the *SaFX* allele in weeds, either as a way to decrease hybridization barriers with the local crop or as a way to circumvent other possible fitness effects of the incompatibility interaction.

*SaFX* may create a new version of a tri-allelic system involved in the evolution of speciation genes. Another wide-compatibility allele has been reported at *Sa*, characterized by two polymorphisms, a 6 bp insertion in *SaM* and a SNP in *SaF* [29]. The occurrence of wide-compatibility alleles in hybrid sterility systems is not uncommon, as these alleles have also been reported at other hybrid sterility loci [31]–[33].

All known wide-compatibility alleles act to restore fertility. Relaxation of sterility barriers can be favored if hybrids with wide-compatibility alleles have higher or equal fitness to their parents. Additionally, as is seen in the killer-protective system at *s5,* some sterility genes do not function directly in pollen or seed production and deleterious interactions between alleles at these loci, while promoting sterility, can cause other problems within the organism unrelated to gamete development (endoplasmic reticulum stress in the case of s5 [34]. However, intra-cellular complications caused by incompatible interactions at *Sa,* besides hybrid semi-sterility, have not been reported, though the fitness effects of the *SaF+*− *SaM* heterozygote interaction have not been fully investigated. Interestingly, in our study, only the hybrid sterility gene(s) that function directly in gamete development (the *DPLs)*, which are implicated in pollen development; [12] did not display a putative wide-compatibility allele.

### The Potential for Weed-Crop Gene Flow

Because US weedy rice groups descend from closely related *indica* and *aus* cultivars, while the local US rice crop is of *japonica* descent, we expected that typical *indica*-*japonica* postzygotic hybrid sterility barriers would occur between these two groups. Surprisingly, examination of three cloned hybrid sterility systems suggests that fewer postzygotic barriers exist between US weeds and the local crop than what is typically observed between *indica* and *japonica* cultivated rice subspecies. Given the prominence of the wide compatibility deletion at the *s5* locus in all weedy groups, there do not seem to be postzygotic barriers decreasing the possibility of gene flow with the local *japonica* crop at this locus (Table 1). Functional alleles at both *DPL* loci predominated in the SH, MX and BRH weed groups, implying that no crosses involving these weed groups can give rise to the sterility causing genotype *DPL1-K−/DPL2-N*− (Table 1). While BHA groups did have a high incidence of nonfunctional *DPL1* alleles, the dearth of nonfunctional alleles at either *DPL* locus in the local *japonica* crop also suggests few barriers to gene flow (Table 1). Few barriers for crop-weed gene flow are also apparent at the *Sa* locus for BRH and SH groups, as *SaFX,* a seemingly wide-compatibility allele, is nearly fixed. *SaFX* is absent in both BHA groups, indicating these groups are less likely to able to hybridize without issues of sterility with the local crop. However, the overall lack of postzygotic barriers to gene flow given the ancestry of US weeds is remarkable, and could suggest that *japonica*-compatible weed types have been favored during weed evolution in the US.

That weed-crop gene flow occasionally occurs in US rice fields is known. As mentioned above, MX weeds are known hybrids of SH or BHA weeds with the local *japonica* crop [16], and we have also found evidence of more localized genomic introgression of *japonica* alleles in some members of the BHA1 group [20]. However, the rate of crop-weed hybridization historically in the US has been low, <1% [13], [19]. Most hybrid incompatibility systems described in rice lead to only partial sterility in hybrids, and are expected to decrease the rate of hybridization, not eliminate it, which could account for low rates of gene flow between crops and weeds. Additional possible postzygotic fitness consequences in F1, such as overly late flowering that could compromise seed set [35], have not been sufficiently explored to draw firm conclusions about their overall contribution to reproductive isolation. In general, our study suggests that genetically based postzygotic barriers to hybridization are almost nonexistent, implying that prezygotic barriers [36] are more likely the main barriers to extensive gene flow between weedy and cultivated rice grown in the US. Possible prezygotic barriers to weed-crop outcrossing include the high self fertilizing levels of both weedy and cultivated rice due to short pollen longevity [37], and differences in flowering time among groups. For example, BHA weeds tend to flower later than SH weeds or the *japonica* crop, although variability in flowering time exists in all groups [20], [38]. Our study also suggests that under the right environmental circumstances or selective pressure, gene flow levels between crops and weeds have the potential to increase.

The use of herbicide resistance (HR) rice cultivars is increasing in the US, and in 2011 60–65% of Southern US rice was reported to be HR [39]. If the hybrid sterility loci used in this study adequately represent how other hybrid sterility loci have evolved within weedy groups, then the capacity of weedy groups to freely cross with these HR cultivars and produce fertile offspring lends itself to the creation of HR red rice, undermining many weed prevention strategies. In this respect, use of HR rice and herbicide applications changes the selective environment for weeds, such that HR hybrid weeds will be selectively favored. Alternatively, gene flow of unfavorable weedy traits, including shattering and dormancy, could be passed into native cultivated fields, which could interfere with uniform harvesting conditions. Likewise, the escape of an HR gene from red rice could also contaminate fields dedicated to non-HR rice [19].

Hybrid sterility is the most common form of postzygotic isolation in plants, and has long been of interest to evolutionary biologists, due to its importance in the speciation process, and to breeders, due to its impact on crop-improvement strategies. The importance of hybrid sterility barriers to crop-weed gene flow has not explicitly been considered, and here we have shown that there is greater potential than expected for crop-weed hybridization in US cultivated rice fields. As planting of HR rice has increased in the southern rice belt over the last 10 years, and reports of herbicide resistant weedy rice begin to surface [40], it is apparent that neither prezygotic nor postzygotic mating barriers are likely to impede weedy rice from acquiring the crop alleles that will enable them to survive in an herbicide-rich environment. Development of effective weed management strategies must take into account the greater than expected capacity for gene flow between weedy rice and its conspecific crop.

## Supporting Information

Table S1 **Core** ***Oryza*** **accessions used in the study.**
(XLSX)

Table S2 **Additional individuals genotyped for** ***s5*** **and** ***SaF*** **.**
(XLSX)

Table S3 **Primers used for amplification and sequencing of the hybrid incompatibility loci.**
(XLSX)

Table S4 **Allele types and haplotypes observed for all individuals in our core set at the hybrid incompatibility loci.**
(XLSX)

Table S5 **Haplotype tables for hybrid incompatibility loci.**
(XLSX)
